# Information can be stored in the human skin memristor which has non-volatile memory

**DOI:** 10.1038/s41598-019-55749-9

**Published:** 2019-12-17

**Authors:** Oliver Pabst, Ørjan G. Martinsen, Leon Chua

**Affiliations:** 1 Department of Physics, University of Oslo, Oslo, Norway; 2 Department of Clinical and Biomedical Engineering Oslo University Hospital, Oslo, Norway; 3 Department of EECS, University of California, Berkeley, Berkeley, CA USA

**Keywords:** Biomedical engineering, Biological physics, Electrical and electronic engineering

## Abstract

Much is already understood about the anatomical and physiological mechanisms behind the linear, electrical properties of biological tissues. Studying the non-linear electrical properties, however, opens up for the influence from other processes that are driven by the electric field or movement of charges. An electrical measurement that is affected by the applied electrical stimulus is non-linear and reveals the non-linear electrical properties of the underlying (biological) tissue; if it is done with an alternating current (AC) stimulus, the corresponding voltage current plot may exhibit a pinched hysteresis loop which is the fingerprint of a memristor. It has been shown that human skin and other biological tissues are memristors. Here we performed non-linear electrical measurements on human skin with applied direct current (DC) voltage pulses. By doing so, we found that human skin exhibits non-volatile memory and that analogue information can actually be stored inside the skin at least for three minutes. As demonstrated before, human skin actually contains two different memristor types, one that originates from the sweat ducts and one that is based on thermal changes of the surrounding tissue, the stratum corneum; and information storage is possible in both. Finally, assuming that different physiological conditions of the skin can explain the variations in current responses that we observed among the subjects, it follows that non-linear recordings with DC pulses may find use in sensor applications.

## Introduction

**Figure 1:**
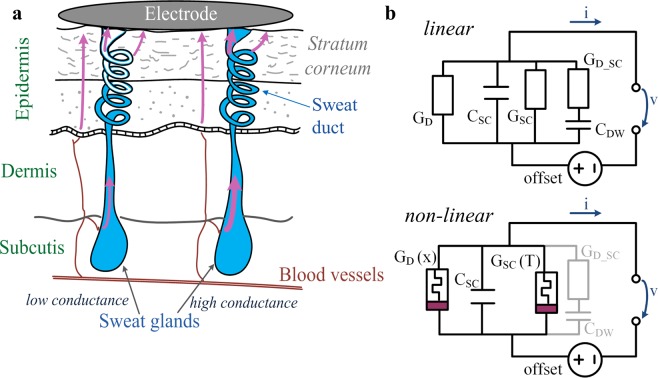
Schematic of human skin, (**a**) showing different layers, sweat glands, blood vessels and possible current pathways through the glands and the epidermis (indicated by the purple arrows). The illustration is not to scale, e.g. the epidermis is actual much thinner than the dermis. The secretory parts of the glands are located within the subcutis and the upper part of a sweat gland which is extending from the dermis to the epidermis up to the skin surface is called “sweat duct”. (**b**) Skin equivalent circuits within the linear (top) and non-linear range (bottom). The ionic pathways through the sweat ducts and the stratum corneum can be modeled by the conductances, *G*_*D*_ and *G*_*SC*_, within the linear range, respectively. If the measurements are within the non-linear range, the ability to conduct currents is dependent on an internal state that is affected by the electrical stimulus. Instead of linear conductances, the ionic pathways through the sweat ducts and the stratum corneum are now modeled by the state dependent conductances *G*_*D*_*(x)* and *G*_*SC*_*(T)* (this model is based on the findings in^1^). The serial connection of the components *C*_*DW*_ and *G*_*D_SC*_ models the frequency dependent component of the conductance in an AC measurement (based on the findings in^26^) and is negligible for the non-linear measurements, using a very low frequency AC or DC voltage as stimulus. The capacitive properties of the stratum corneum are related to its humidity^28^ and modelled by the component *C*_*SC*_ in both, linear and non-linear, equivalent circuits.

This study can be placed within the field of non-linear electrical measurements on human skin (see also^1^). An electrical measurement is non-linear if the measured phenomenon changes with the amplitude of the excitation, i.e. if it violates the principles of superposition^2^. The waveform of the recorded signal is different from the applied stimulus. Non-linear electrical measurements reveal the non-linear electrical properties of a material and can be done with low frequency alternating current (AC) or direct current (DC) electrical stimuli of high amplitude or DC level. As the frequency increases or amplitude and DC level decrease, the measurement will become more linear. Non-linear electrical properties have been demonstrated for human skin (more specifically for the sweat ducts) in^3^ (using a constant amplitude voltage stimulus) and in^4^ (using a constant amplitude current stimulus). These properties were explained by electro-osmosis (directed motion of liquids forced by an electric field) that changes the degree of sweat duct filing^3^. If the sweat (which contains ions) reaches the skin surface, well conductive current pathways through the sweat ducts are provided (see illustration in Fig. 1a). At low frequency AC or DC stimuli, these pathways dominate the measurements. Non-linear properties have also been demonstrated for the stratum corneum surrounding the sweat ducts^5^. It was later concluded that the sweat ducts (see^6^) and the stratum corneum (see^1^) can both be modelled as memristors (both are electrically in parallel to each other). A memristor (**mem**ory **r**es**istor**) is often labelled as the fourth passive electrical circuit element^7^ and its “fingerprint” is a pinched hysteresis loop in the voltage current plot with pinched point in the origin of coordinates^8^. Unlike a resistor, a memristor has an internal state that may change with an applied electrical stimulus. Voltage and current are related via the state-dependent Ohm’s law which is given as

1 $$v=M(x)i$$

2 $$\frac{d{\boldsymbol{x}}}{dt}=f({\boldsymbol{x}},\,i)$$

for generic memristors^9^ (like human skin^1^) using memristance *M*(***x***) (state dependent resistance) with ***x*** as a vector of state variables. A memristor can also be described by its state-dependent conductance (“memductance”) which is useful in parallel circuits. Different realizations of memristors have been presented, for example, in^10^ (based on titanium dioxide), in^11,12^ (based on tantalum oxide) and in^13^ (based on zinc oxide). Beside human skin, other biological memristors have been found, for example, in the Venus flytrap^14^ and in slime molds^15^. There are possible applications of memristors in circuits such as for neuromorphic computing^16–18^, emulating arithmetic operations^19,20^, and for frequency doubling^21^ or rectification of electrical currents^22^.

However, regardless of the term “memory”-resistor, the storage of information is only possible with non-volatile memristors. A passive memristor (i.e. no internal batteries) is non-volatile if there exist at least two distinct stable equilibrium states, which do not diverge from any infinitesimal disturbance^23^. The volatility of a 1^st^-order memristor with a strictly monotone-increasing memductance can be tested by non-linear measurements; if the steady state current responses to periodic DC voltage pulses are periodic, the underlying memristor is volatile, otherwise it is non-volatile^23^. We assume, without loss of generality, as in sections 6 and 7 of^23^, that the memductance function G(x,v) is a strictly monotone-increasing function of x, for fixed voltage v. The converse result for a strictly monotone-decreasing function, follows, *mutatis mutandis*.

Here we studied whether the human skin memristor is volatile or non-volatile by applying three series of DC voltage pulses to the test subjects (28 in total). Recordings were done at the forehead, the earlobe and the fingertip. The here chosen DC levels (−0.8 V and 0.8 V) were sufficient to cause non-linear measurements (see for example the predication of the range of linear vs. non-linear measurements at the forehead in Fig. 3 in^1^). The recorded currents changed with each DC pulse but the state of the skin memristor also changed after the voltage was turned off.

We studied the latter further by recording the small signal conductance for three minutes after each pulse series. The excitation was done with a sinusoidal voltage with a frequency of 20 Hz and amplitude of 0.1 V. This measurement method is linear since the applied voltage does not affect the state of the skin memristor and is also suitable for the recording of electro dermal activity (see also^24,25^); i.e. for the recording of changes in sweating stimulated by sympathetic and parasympathetic nerve activity that can be mainly observed in the palms (the fingertip is part of it) and plantar skin sites.

Electrical equivalent circuits for linear and non-linear measurements on human skin are given in Fig. 1b. Linear conductance components representing the sweat ducts and the stratum corneum within linear measurements are replaced by state dependent conductance components within the non-linear measurements. Recorded currents are usually larger if galvanic contact through the sweat ducts was achieved which means that the sweat duct memristor is more or less dominating the measurements in these cases^1^. However, there are cases in which both, the sweat duct and the stratum corneum memristor contribute noticeably to the measurement.

The conductance obtained from a recording with a 20 Hz sinusoidal voltage is slightly higher than that obtained from a recording with a DC voltage (see^26^) due to a frequency dependent conductance component. A current pathway from the sweat duct passing through the sweat duct wall and finally going through the stratum corneum or the epidermis (modelled by the serial connection of the C_DW_ and G_D_SC_ in Fig. 1b) would be a possible explanation for this frequency dependent conductance component. A DC current will not pass through the sweat duct wall.

We suggested^1^ to allocate non-linear electrical measurements on human skin (and biological tissues in general) within the field of Bioimpedance^27^ and suggested the terms “non-linear Bioimpedance”, “state-dependent Bioimpedance” or “Bio**mem**pedance” if the non-linear part needs to be stated explicitly. The finding that the non-linear electrical behavior of human skin falls under the classification of a memristor enables us to use the associated models for analyzing and modeling these properties.

## Results

**Figure 2:**
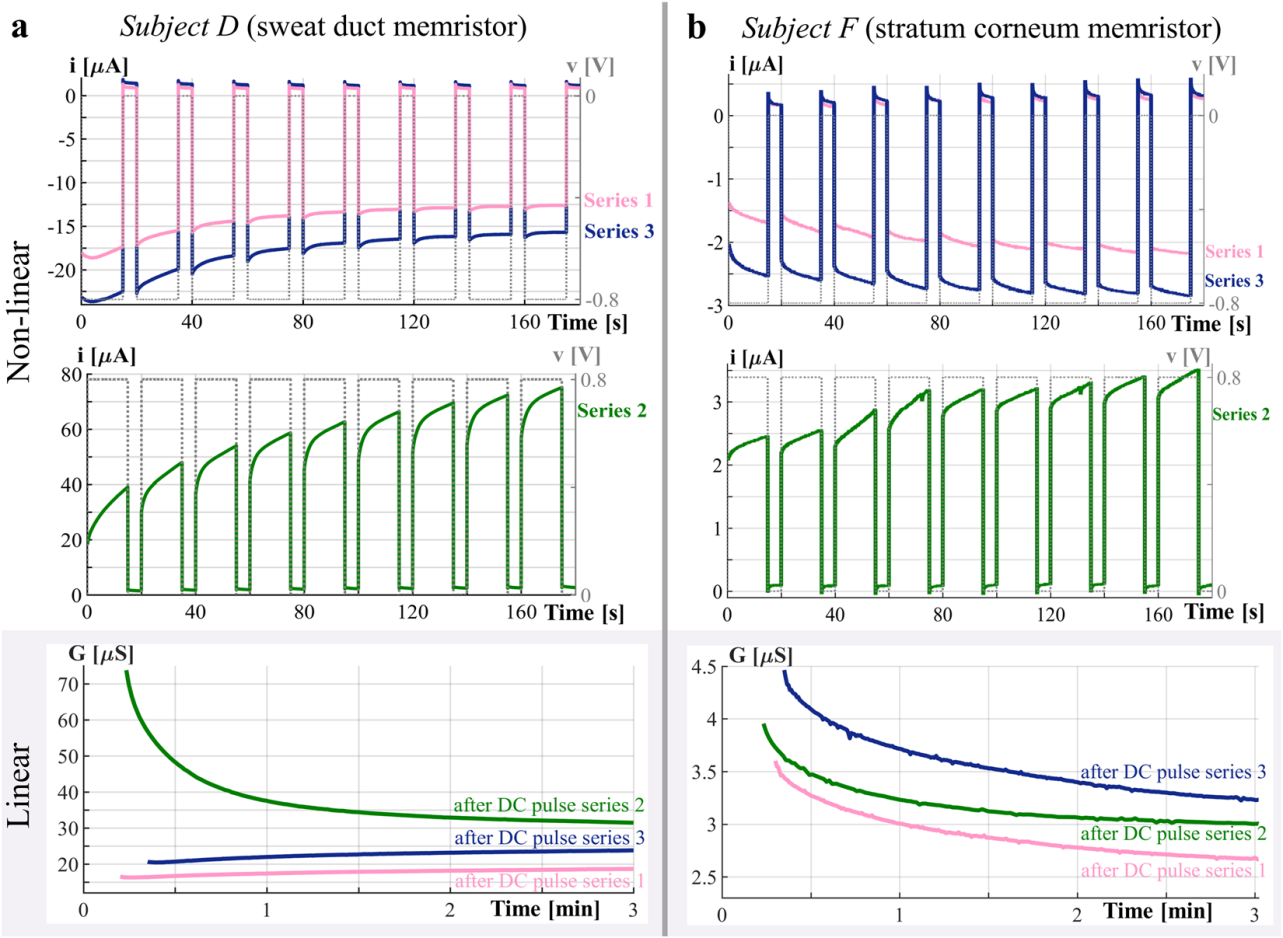
Recordings from the forehead shown for two different subjects, (”D” and “F”, same notification as in^1^). The results (presented as current, *i*, and voltage, *v*, over time) of the first and third pulse series (pulse height was −0.8 V for both) are combined in the top plot, the results of the second pulse series (pulse height of +0.8 V) are shown in the middle plot. The small signal (linear) conductance measurements after each DC pulse series are combined in the bottom plot (presented as conductance over time). (**a**) Results from subject “D”. The measurements are dominated by the sweat duct memristor. This subject exhibited one pinched point in the AC voltage-current plot (see Supplementary Fig. S5) and the change of the state dependent conductance depends on the polarity of the applied voltage as it can be seen here. The small signal conductance states (see bottom plot) are very different even after three minutes of recovery. (**b**) Results from subject “F”. The measurements are dominated by the stratum corneum memristor. This subject exhibited two pinched points in the AC voltage-current plot (see Supplementary Fig. S5) and there is an increase in the state dependent conductance independent of the polarity of the applied voltage as it can be seen here. There are also differences in the small signal conductance states that can be observed even after three minutes of recovery (see bottom plot).

**Figure 3:**
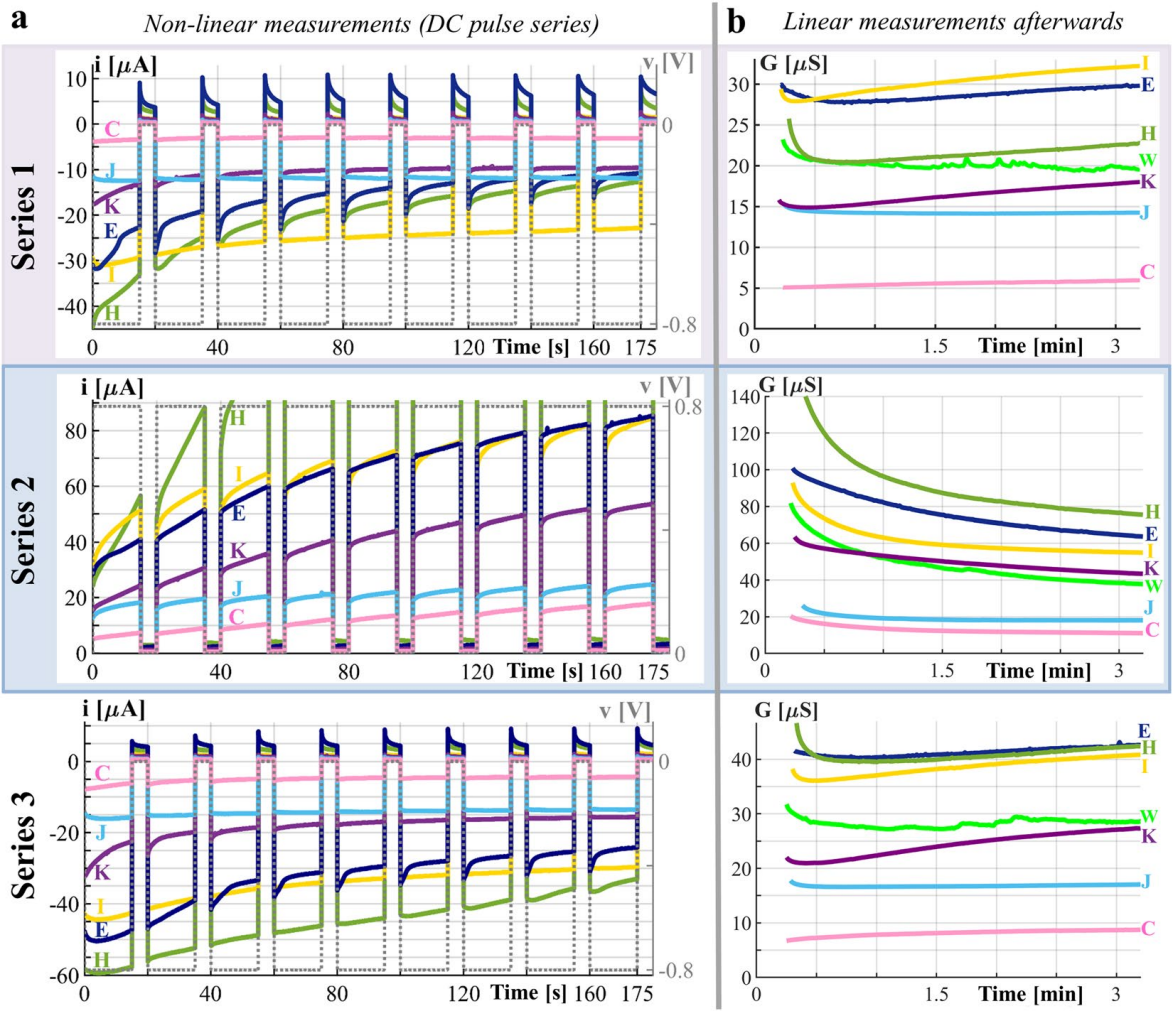
Example recordings from the forehead, shown for subjects C, E, H, I, J and K. (**a**) Non-linear measurements with DC voltage pulse. Measured current, *i*, and applied voltage, *v*, plotted over time. The limit of the y-axis in series 2 is set to 91 µA. Subjects C, K, E, I, H reflect the sweat duct memristor since the amount of current decreases within series 1 and 3. The current of subject H, which is an outlier, reaches 171.5 µA at the end of the last pulse (the corresponding memductance is 214 µS). (**b**) Small-signal conductance measurements after each DC pulse series (shown for the same subjects). The time is related to the end of the last pulse of the corresponding DC pulse series. The limit of the y-axis in series 2 is set to 140 µS. The results for an additional subject, W, show that the skin at the forehead may be emotionally active in some subjects.

**Figure 4:**
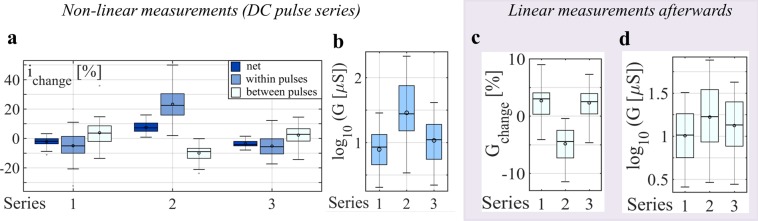
Boxplots based on the recordings from the forehead, including all (*N* = 28) test subjects. The horizontal line in the middle of each boxplot denotes the median; the circle indicates the mean value; and the whiskers indicate the 5% and 95% percentiles. (**a**) The average changes in current, *i*, within pulses (within 15 seconds) and between pulses (within 5 seconds) and the average net change from pulse to pulse are presented as ratios for all three series. The memductance of many but not all subjects changed noticeably between pulses (compare, e.g., subjects E and I in Fig. 3a). The measured current (and consequently memductance) in, for example, series 1 increased by 3.6% between each pulse and decreased on average by 5.1% within each pulse (median values). A memductance decrease of 2% was observed with each pulse (net change). The changes in memductance in series 2 occurred faster. A decrease between pulses of 9% (median), an increase within pulses of 22.5% and a net increase of 7.2% with each pulse were observed. (**b**) Memductance *G* (logarithm to base 10) at the end of the last pulse (at 175 s) of each series. The memductance values of the 25% and 75% percentiles after series 2 are around 15 µS and 75 µS. The memductance values of the 25% and 75% percentiles after series 1, for example, are around 4.5 µS and 13.3 µS, respectively. (**c**) Change in the small signal conductance from minute 2 to minute 3 (after the last pulse in each pulse series). (**d**) Small-signal conductance *G* (logarithm to base 10) 3 minutes after the last DC pulse. Results from the Friedman test show that there are significant differences in the conductance values (logarithm to base 10) among the three groups (p-value < 0.001, chi-square = 52.286 with 2 degrees of freedom, number of subjects is 28). Pairwise comparison (Tukey Test with a p-value < 0.05) shows that all groups differ from each other.

The galvanic contact through the sweat ducts was usually achieved at the forehead (see data in Figs. 2–4, and Supplementary Fig. S5) and most of the recordings (25 out of 28 subjects) were dominated by the sweat duct memristor. Three out of 28 subjects reflect the stratum corneum memristor. More data from the stratum corneum memristor (18 out of 28 subjects) was obtained from the earlobe (see Supplementary Figs. S1–S3) since galvanic contact through the sweat ducts was only achieved from 9 out of 28 subjects (compare also with^1^).

### Sweat duct memristor

Within the sweat duct memristor, the sweat is moved by electro-osmosis^3,6^ towards the skin surface or towards deeper skin layers dependent on the polarity of the applied stimulus. There will be an increase in the state dependent conductance in the former case and a decrease in the latter case. This behavior can be observed from subject D in Fig. 2a (as well as, from subjects C, E, H, I, K in Fig. 3, subjects D, L, G, and N in Supplementary Fig. S1, and subject T in Supplementary Fig. S5). These are the same subjects that exhibited hysteresis loops with only one pinched point in the AC voltage current plots (see subjects D and T in Supplementary Fig. S5). With a positive DC level, the measured current increases from pulse to pulse in series 2 which implies that the sweat is pulled towards the skin surface. With negative DC pulses (series 1 and 3), the sweat was pushed inwards, towards deeper skin layers, and the memductance consequently decreased. The peak of the current response changes after each additional voltage step, which implies a cumulative change in memductance. However, the differences in the peaks become smaller with each pulse. The measured currents at the end of one pulse and the beginning of the next pulse are usually different from each other which means that the memductance value does not stabilize as the voltage drops to zero. Within the small signal conductance measurements after each pulse series (Fig. 2a, bottom) there is a continuous decrease in the conductance after series 2 and an increase within the 3 minutes of measurement after series 1 and 3.

There are large differences between subjects in how the memductance changes with the applied voltage but also after the voltage is switched off (see Fig. 3). Subject H, for example, exhibited large changes in current caused by the applied voltage. Between pulses in series 2 the decrease in conductance of subject H is quite large as it is for subject I. The memductance of both subjects keeps quite constant between pulses in series 1 and 3. The changes in memductance between pulses of subject E on the other hand are relatively small in series 2 but quite large within pulse series 1 and 3.

The qualitative evaluation of the forehead recordings over all subjects (see Fig. 4) reflects mainly the sweat duct memristor. A cumulative decrease (from pulse to pulse, net change in Fig. 4a) in the amount of current (and consequently in state dependent conductance) can be observed for most subjects if the applied DC level is negative (Series 1 and 3). If the applied DC level is positive (Series 2), there is a cumulative increase in the amount of current and the state dependent conductance at the end of series 2 is much higher as it is after series 1 and 3 (see Fig. 4b). Even two to three minutes after the non-linear measurement with DC pulses, the small signal conductance has not stabilized (see Fig. 4c) and increases for most subjects after negative pulses have been applied (series 1 and 3) and decreases for all subjects after the series with applied positive DC pulses (series 2). However, the small signal conductance values at minute three after the last DC pulse of each series are still statistical significant different from each other (Fig. 4d).

### Stratum corneum memristor

Within the stratum corneum memristor, the change in the state dependent conductance is related to heating^5^ of the keratinized tissue. The state dependent conductance increases as current passes through independent of the polarity of the applied stimulus. This behaviour can be observed from subject F in Fig. 2b (as well as, from all the subjects in Supplementary Fig. S2, and subject U in Supplementary Fig. S5). These are the same subjects that also exhibited two pinched points within the AC voltage current plots (see subjects F and U in Supplementary Fig. S5). The amount of current increases (as the memductance does consequently) within each pulse in series 2. However, the same occurs also when negative pulses are applied (series 1 and 3), which is different from the recordings that are dominated by the sweat duct memristor (compare Fig. 2a with b). Similar to the sweat duct memristor, the measured currents at the end of one pulse and the beginning of the next pulse are usually different from each other. The heat in the stratum corneum that is caused by the electric current under the measurement electrode will be distributed among the surrounding tissue and the temperature decreases. When there is no current (between pulses) the memductance decreases as a consequence. This can be also confirmed by the small signal conductance measurements after each series (see Fig. 2b bottom). A continuous decrease in the small signal conductance was observed after each pulse series which is different from the sweat duct memristor recordings.

Variations in the (state dependent) conductance between subjects are smaller than it was for the sweat duct memristor which is revealed from the earlobe recordings (see Supplementary Fig. S2). The amount of current increased independent of the polarity of the applied DC pulses for almost all subjects and decreased in absence of the applied voltage (see Supplementary Fig. S3a bottom); However, there are quite large variations between subjects to which extend these changes happen. The overall net change in the amount of current is positive for almost all subjects (independent of the polarity of the applied pulses) but close to 0% which gives indication that the obtained current responses are quite symmetric. Compared to the sweat duct memristor, less variation in the state dependent conductances (among the series 1, 2, and 3) after the last DC pulses (see Supplementary Fig. S3b bottom) and the linear conductances (see Supplementary Fig. S3d bottom) are observed.

The electrical properties of the stratum corneum usually dominate the measurements when there is only little or no galvanic contact through the sweat ducts. Corresponding currents are quite small and the memductance of subject F in Fig. 2b, for example, ranges from about 2.5 µS to 5 µS. However, the stratum corneum of some subjects may be even better conducting as it is for subject U in Supplementary Fig. S5 whose memductance ranges from about 3.75 µS to 12.5 µS. The recording of subject T (Supplementary Fig. S5) is dominated by the sweat duct memristor and the memductance is quite low compared to other recordings that are dominated by the sweat duct memristor. However, both subjects demonstrate that the memductance of the stratum corneum and the sweat duct memristor can obtain values within the same range. Subject J (Fig. 3) is an example in which both memristors contribute noticeably to the measurement. It exhibited an increase in memductance during series 1. In series 3, slight increases in memductance within most of the pulses are observed. However, its memductance at the end of series 3 is slightly smaller than it was in the beginning of this series.

## Discussion

**Figure 5:**
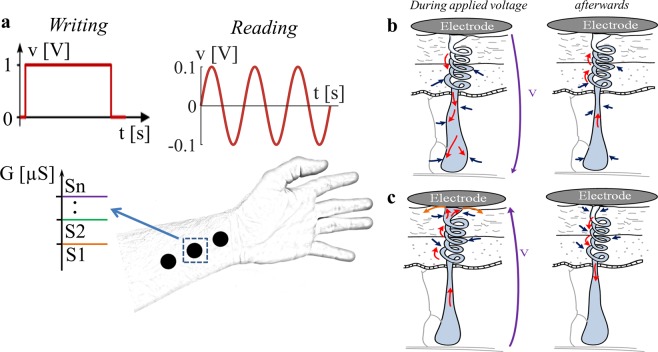
Illustration of the discussion. (**a**) Illustration of storing and reading of information in human skin. Information writing can be done by a DC voltage with a level of, for example, 1 V. Different conductance states can be set by variation of the duration of the DC pulse. The reading can be done by a small signal conductance measurement. The interpretation as state S1, S2, …, or Sn is based on the recorded conductance. (**b**) and (**c**) Illustration of change in sweat level in a single sweat gland/duct while a voltage, *v*, is applied and released afterwards. The here shown illustration is based on the findings from applied DC pulse series (Figs. 2 and 3). Red arrows indicate possible ion movements, while blue arrows indicate possible pressure driven counter-forces that build up when the volume of the sweat gland/duct increases. (**b**) (left) The direction of the voltage is from skin surface towards deeper skin layers. The sweat is pushed towards deeper layers, resulting in a memductance decrease as well as an increase of the sweat gland/duct volume within the deeper layers. (right) Resulting counter-forces will move the sweat back towards the skin surface as soon as the voltage is released. (**c**) (left) The direction of the voltage is from deeper skin layers towards the skin surface. The sweat is pulled towards the skin surface resulting in a strong memductance increase. Sweat that reaches the skin surface may diffuse into the surrounding tissue (illustrated by orange arrows). (right) Pressure driven forces will push the sweat back towards deeper skin layers as soon as the voltage is released.

The current responses (obtained from the forehead and the earlobe) to periodic constant voltage steps (i.e., a square wave) are not periodic which is indication that the underlying memristors are non-volatile. This is valid for both, the sweat duct memristor (see Figs. 2a, 3, and Supplementary Fig. S1) and the stratum corneum memristor (see Fig. 2b and Supplementary Fig. S2). Except, possibly, for brief initial transients due to parasitic capacitance, our measurements show the current pulses increase monotonically, as in Figs. 48 and 50 (top) of ^23^. The change in memductance occurs fast within the first few seconds of each pulse and then rapidly slows down, and it also occurs after the voltage was turned off. Since the memductance changes in absence of an applied voltage happen quite fast (within 5 seconds between pulses) it is likely that a counter force driven by pressure from the surrounding tissue drives the sweat back (see illustrations in Fig. 5b,c). Furthermore, the applied voltage creates differences in ion concentrations, and the gradient-driven diffusion processes will cause a change as well as reabsorption processes of sweat into the surrounding tissue. With longer relaxation times, i.e. pauses between pulses (e.g. 1 minute instead of 5 seconds) the state dependent conductance might have obtained similar values at the beginning of each pulse for some subjects. However, the authors think that the current responses would still have been non-periodic. First, there are test subjects that seem to experience (almost) no memductance change between pulses (see, for example, series 1 and 3 of subject I in Fig. 3). All these subjects also exhibited non-periodic current responses, as they would have done with much longer duration between pulses. Second, the significant differences in the small signal conductance after three minutes are a confirmation that the underlying memristors have several stable equilibrium states and thus are non-volatile. The here conducted experiment might be repeated with longer pulse duration and longer pauses between pulses.

It is actually possible to store information in human skin (analog memory) for at least three minutes (Figs. 2, 3b and 4), if not hours. This means that a change in memductance caused by excitation in the non-linear range (e.g. by the used DC voltage pulses) can still be read after three minutes by linear measurements. Different from a binary memory, the human skin memory has more than two distinct states. In subject I (Fig. 3b), for example, it is possible to assign conductance values between 25 µS and 35 µS as state one, values between 35 µS and 45 µS as state two and so on (see also illustration in Fig. 5a). Some physiological parameters like the number of sweat ducts and the skin thickness will not change at the exact same skin site of one test subject. It is therefore likely that the same definition range and number of states can be used for repeated trials. However, there are large differences in the recordings among the subjects, which means that the range of each state (and the number of possible states) has to be defined specifically for each subject (and skin site).

The change in conductance slows down with time. Some subjects like E and J (Fig. 3b) demonstrate that information storage is possible in principal for much longer than three minutes since the measured conductances stabilize more or less around different values (equilibrium states). This finding may have significant implications for medical, health, and sport applications, in which non-volatile, analogue memory in the skin may be exploited for wearable sensors and wireless body area networks.

The different equilibrium states may emerge from several physiological processes. If the sweat reaches the skin surface, it will add moisture to the stratum corneum surrounding the sweat duct. This will cause a slight increase in the small signal conductance and susceptance^28^ recorded by the sinusoidal voltage (*f* = 20 Hz) but has a negligible effect on the DC current responses within the pulse series. However, the higher the moisture content in the stratum corneum, the less sweat can diffuse into it from the duct, potentially causing more sweat to remain in the sweat duct itself and even when the sweat is pushed inwards by an electric field, different equilibrium states seem to emerge at the end of series 1 and 3 (see, for example, subjects K or I in Fig. 3a).

It seems that information storage is possible in the stratum corneum memristor as well, since there are significant differences in conductance three minutes after series 1 and 3 (see Supplementary Fig. S3d bottom). However, as it can be seen, for example, from subject F in Fig. 2b, these differences are quite small. Since there is an increase in the state dependent conductance, independent of the applied voltage, it will not be possible to reduce the conductance with the applied voltage.

Any thermal sweating (e.g. caused by physical exercise) will change the state of the sweat duct memristor, which will erase or at least modify any stored information. Humidity and temperature of the environment are likely to have an effect on the measurements in this regard. In a warm and humid environment, the test subjects will experience thermal sweating and thus well conducting current pathways through the sweat ducts would be provided as the sweat reaches the skin surface. It is possible, for example, that the galvanic contact through the sweat ducts at the earlobe would have been achieved from more test subjects. The results can be very different just because of the amount of the initial sweating, for example, before and after physical exercise^29^. It could be useful to add physical exercise to the protocol in the beginning of the test session to somehow standardize the measurements and to increase the galvanic contact (at least if the sweat duct memristor is the subject of interest). In addition, one has to be aware that a non-linear electrical measurement can affect measurements that are conducted short time afterwards.

The sweat duct memristor is also present in the fingertip but any contribution of the stratum corneum memristor (if existent) to the measurement is negligible^1^. However, the current measured during the pulse series (Supplementary Fig. S4) did not follow a clear tendency, and these measurements were obviously influenced by emotional sweating. Therefore, it is not clear from the results here whether the sweat duct memristor at the fingertip is volatile or non-volatile and whether information could be stored.

The recording of subject W in Fig. 3b implies that emotional sweating can also occur at the forehead and may also slightly influence the non-linear measurements at this skin site. On the other hand, increasing the sweat level by electro-osmosis may also enable the recording of emotional sweating at other than palmar and plantar skin sites.

The small signal conductance measurements with applied sinusoidal voltage with amplitude of 100 mV and frequency of 20 Hz are linear^1^. The time is too short and the amplitude too low to have any noticeable changes in the memductance from ion movements in the sweat ducts. The heating of the stratum corneum is independent of the sign and could in principle occur within the small signal conductance measurements. However, it is very likely that the heating due to an applied sinusoidal voltage with an amplitude of 100 mV (the rms value is 70.7 mV) is negligible. The thermal dissipation is described by P = V^2^·G and if we assume the conductance of the stratum corneum, for example, to be 10 µS (it was usually below), then the dissipated power will be 50 nW for the applied sinusoidal voltage. In comparison, it is 6.4 µW for the applied DC voltage pulses with a level of 0.8 V.

The choice of the signal frequency for the linear conductance measurements is quite free. However, the frequency should not be too low (for example, below 1 Hz) since the measurement might be non-linear then. On the other hand, if the frequency is too high (above 1 kHz) the measurement is not dominated by the sweat ducts and the stratum corneum any longer. Frequencies close to the frequency of the mains (which is 50 Hz in Europe) should also be avoided.

## Methods

### Subject recruitment, approval

A total of 28 test subjects (16 male, 12 female, mean age 31 years, SD = 9.5 years) were recruited and gave informed consent for participation in the study. A 29^th^ test subject was recruited but the subject’s skin was initially covered by body lotion, and the collected data were excluded from further analysis. The research related to human use has been complied with all the relevant national regulations (including the Regional Committees for Medical and Health Research Ethics), institutional policies and in accordance with the tenets of the Helsinki Declaration. The research group was responsible for ensuring electrical safety for the test subjects. The measurements were conducted at the University of Oslo in November and December in 2016.

### Experimental design

The here presented experiment is the second out of three from an overall test session (that lasted about one hour) and was performed directly after the first experiment. It was randomly chosen whether the preferred or non-preferred hand side was used for the recordings. Electrodes were put in place before the experimental series started, and a test measurement was performed. If only noise was measured in one channel, the corresponding electrode was re-attached. The average relative humidity and room temperature were measured before the first experiment started and were 30.6% (SD of 5.1%) and 21.6 °C (SD of 0.8 °C), respectively. Different AC voltage stimuli were applied to the skin of the test subjects within the first experiment (duration of about 20 minutes) and the results were presented in^1^.

**Figure 6:**
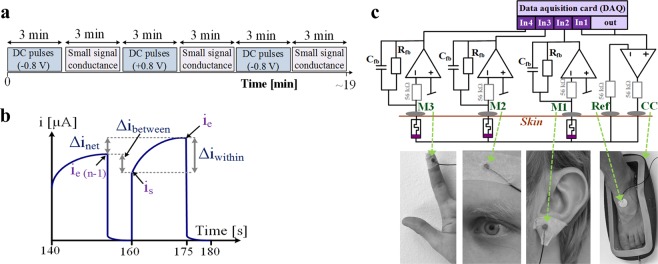
Methods. (**a**) Time schedule of the experiment. (**b**) Schematic representation of the measured current in the last 2 DC pulses of a series and measures that were used for the parameterization. (**c**) Schematic representation of the recording system (top) and corresponding electrode placement (bottom). The three electrode setup (see^30^) with “CC” as the current carrying electrode and a reference (“ref”) electrode enables monopolar recordings under the measurement electrodes (M1, M2, M3); the voltage is basically applied from deeper layers of the skin (under the M electrodes) to the skin surface since the tissue under the CC electrode does not contribute to the measurement. The recordings under the three measurement electrodes were done simultaneously. The data acquisition card USB-6356 from National instruments was used and controlled by custom made software written in NI LabVIEW (version 2014). This is the same setup as presented in^1^ under Creative Commons Attribution 4.0 International License. It was randomly chosen whether all electrodes were placed at the left hand side (shown here) or the right hand side (placement was equivalent).

The here conducted experiment consisted of three series of applied DC voltage pulses, each followed by small signal conductance measurements (see time schedule in Fig. 6a). The voltage steps were always from 0 V to −0.8 V (first and third series) and 0 to +0.8 V (second series). Each single pulse had a duration of 15 seconds in high state, followed by 5 seconds in low state (0 Volts). A continuation of 9 pulses was applied; the pulse series stopped automatically after the 9^th^ pulse and the total duration per series was exactly three minutes. Small signal conductance was measured for about three minutes after each DC pulse series and this recording mode was stopped manually as well as the change between recording modes was performed manually. The recordings of the small signal conductance started around 10 seconds (median time, 5% quartile was 7 seconds, 95% quartile was 16 seconds) after the pulse series were finished.

### Instrumentation

The exact same custom-built measurement system (see Fig. 6c) based on a data acquisition card (DAQ) and electrode placement was used here as it was in^1^ (please see the “instrumentation” and the “Electrode placement” sections in the methods part in^1^ for further information). To ensure physical separation between the mains and the test subjects, the personal computer, monitor and the DAQ were powered by an international medical isolation device (IMEDe 1000 from Noratel AG, Germany). All electrodes were placed on the same side of the body to avoid current paths through the heart. The measurement electrodes (M1, M2 and M3) were prewired, dry Ag/AgCl electrodes (From Wuhan Greentek PTY LTD), with an active area of 0.283 cm^2^, and were taped to the skin. The electrodes were cleaned with ethanol for reuse. One was placed at the earlobe and another at the fingertip of the pointing finger. The third measurement electrode was placed at the forehead above the iris of the eye of the chosen side, at approximately the width of two fingers above the eyebrow. A saline solution was used as a large current-carrying electrode (CC) and the foot was placed in it. A prewired Ag/AgCl electrode, initially covered with solid hydrogel (Type: Kendall 1050NPSM, active area of 5.05 cm^2^) was used as a reference electrode and placed on the top of the foot, which was not covered by the saline solution. More information about the electrode choice can be found in^29^.

Within the non-linear measurements with DC pulses, signal generation and reading were performed with 500 samples per period. The small signal admittance measurements, from which the small signal conductance was obtained after each pulse series, were performed with a sinusoidal voltage with an amplitude of 100 mV and a frequency of 20 Hz. The admittance can be separated into the real part (conductance) and the imaginary part (susceptance) via the lock-in technique^27^, see also^26^. The instrumentation is capable of recording approximately two conductance values per second.

### Parameterization and statistical analysis

The memductance state at the end of each series and the average current changes between pulses are used as quantitative measures. These measures are based on the current measures illustrated in Fig. 6b. Each period of the pulse series is recorded with 500 samples and consists of 15 seconds in which the pulse is applied followed by 5 seconds relaxation. The measures *i*_*s*_ and *i*_*e*_ are the current values at the beginning (at sample 3 of each new period) and end (sample 375) of each pulse. The relative change between pulses is calculated by the difference −Δ*i*_*between*_ divided by *i*_*e*_(*n* − 1); the relative change within pulses is calculated by Δ*i*_*within*_ divided by *i*_*s*_; and the relative net change is calculated by Δ*i*_*net*_ divided by *i*_*e*_(*n* − 1). The average values of those measures were taken for all pulses of one series.

The state dependent conductance, *G*, at the end of the last pulse of each series is calculated from the corresponding current value, *i*_*e*_, divided by the voltage level of the DC pulse. It is log transformed (to the base 10) to decrease the skewness.

The small signal conductance values at exact two minutes and three minutes after the last pulse of the series switched to 0 V were used for the parameterization; the change in conductance (see Fig. 4c and Supplementary Figs. S3c and S4c) was calculated by

3 $$\frac{{G}_{change}}{ \% }=\frac{G(t=3\,min)-G(t=2\,min)}{G(t=2\,min)}\cdot 100,$$

with *G*(*t* = 3 min) and *G*(*t* = 2 min) as the conductance values at minutes three and two. The conductance value at minute three was log transformed (to the base 10) for the representations shown in Fig. 4d and Supplementary Figs. S3d and S4d.

The statistical analysis on the small signal conductance states was performed separately for the three different skin sites by the use of SigmaPlot (version 11). The repeated measures ANOVA on ranks (Friedman test) and the Tukey test for pairwise comparison were used for non-normal or heteroskedastic data. One way repeated measures ANOVA and the Holm-Sidak method for pairwise comparison were used for normal and homoscedastic data.

## Supplementary information

Supplementary Information

## Data Availability

The recorded data have been deposited with figshare. These data can be obtained free of charge from https://figshare.com/s/0cc020188bb610e2ba55.
