# Supplementary material for: Information can be stored in the human skin memristor which has non-volatile memory
Source: Sci Rep. 2019 Dec 17;9:19260. doi: 10.1038/s41598-019-55749-9 (PMC6917753; doi:10.1038/s41598-019-55749-9)
Supplement: Supplementary file 1 — Supplementary Information [file 41598_2019_55749_MOESM1_ESM.pdf]

Supplementary information

Information can be stored in the human skin memristor which has non-volatile memory

Oliver Pabst, Ørjan G. Martinsen and Leon Chua

Corresponding author: Oliver Pabst
Email: oliverpa@mail.uio.no

This PDF file includes: Figs. S1 to S5

Data from the Earlobe

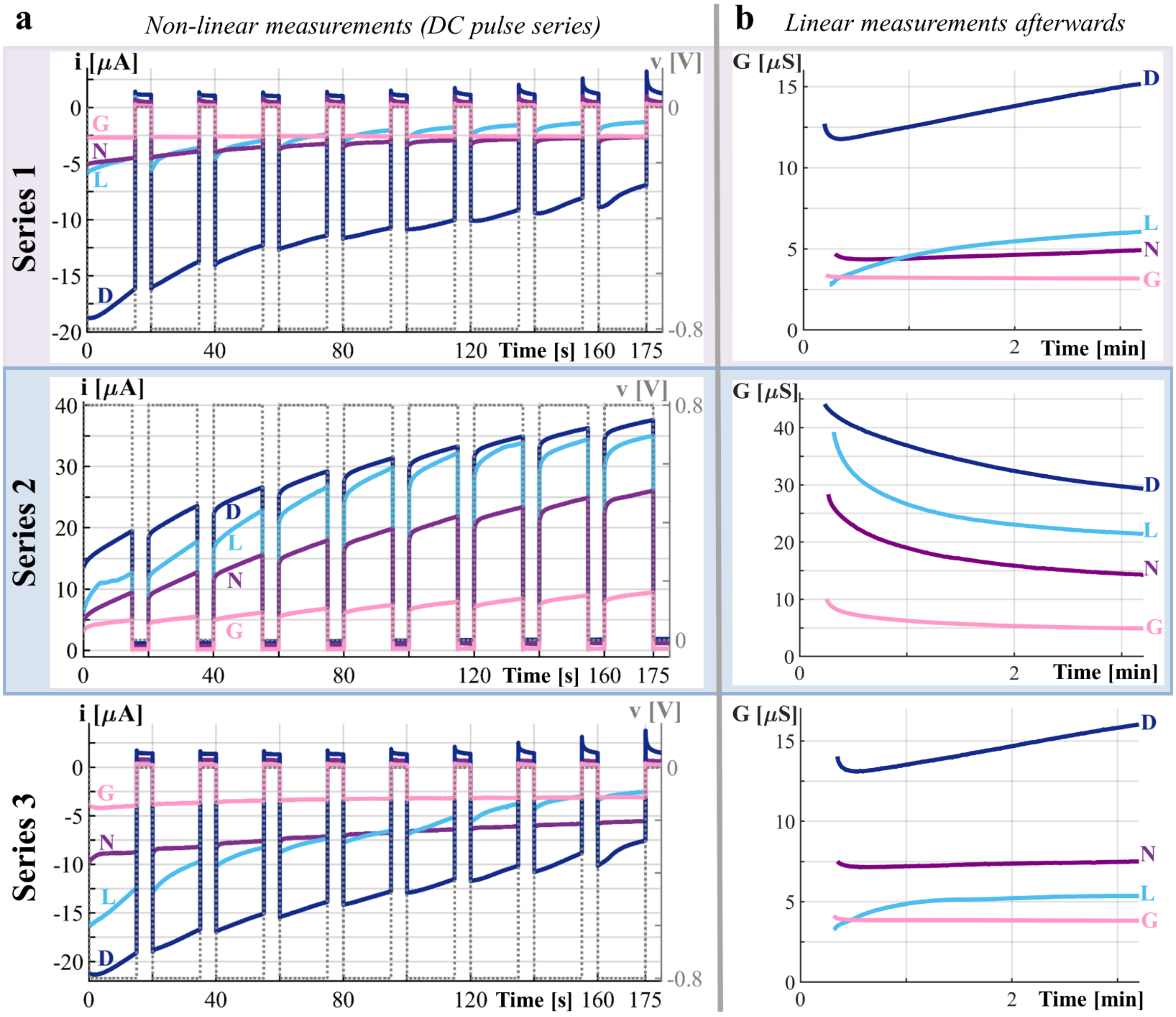

Fig. S1 | Example recordings from the earlobe that represent the sweat duct memristor shown for subjects D, G, L, and N. (**a**) Non-linear measurements with DC pulses. Measured current, *i*, and applied voltage, *v*, plotted over time. The levels of the measured currents and the changes in the currents were in general smaller than that of the forehead. (**b**) Small-signal conductance measurements after each DC pulse series. The time is related to the end of the last pulse of the series. After series 1 and 3, the conductance of some subjects increases following a small decrease in the beginning (subject D and N). Other subjects show an immediate increase (subject L). The conductance of subject G decreases slightly but keeps almost constant. The conductance values of all subjects after series 2 continuously decrease. The decrease in conductance slows down with time.

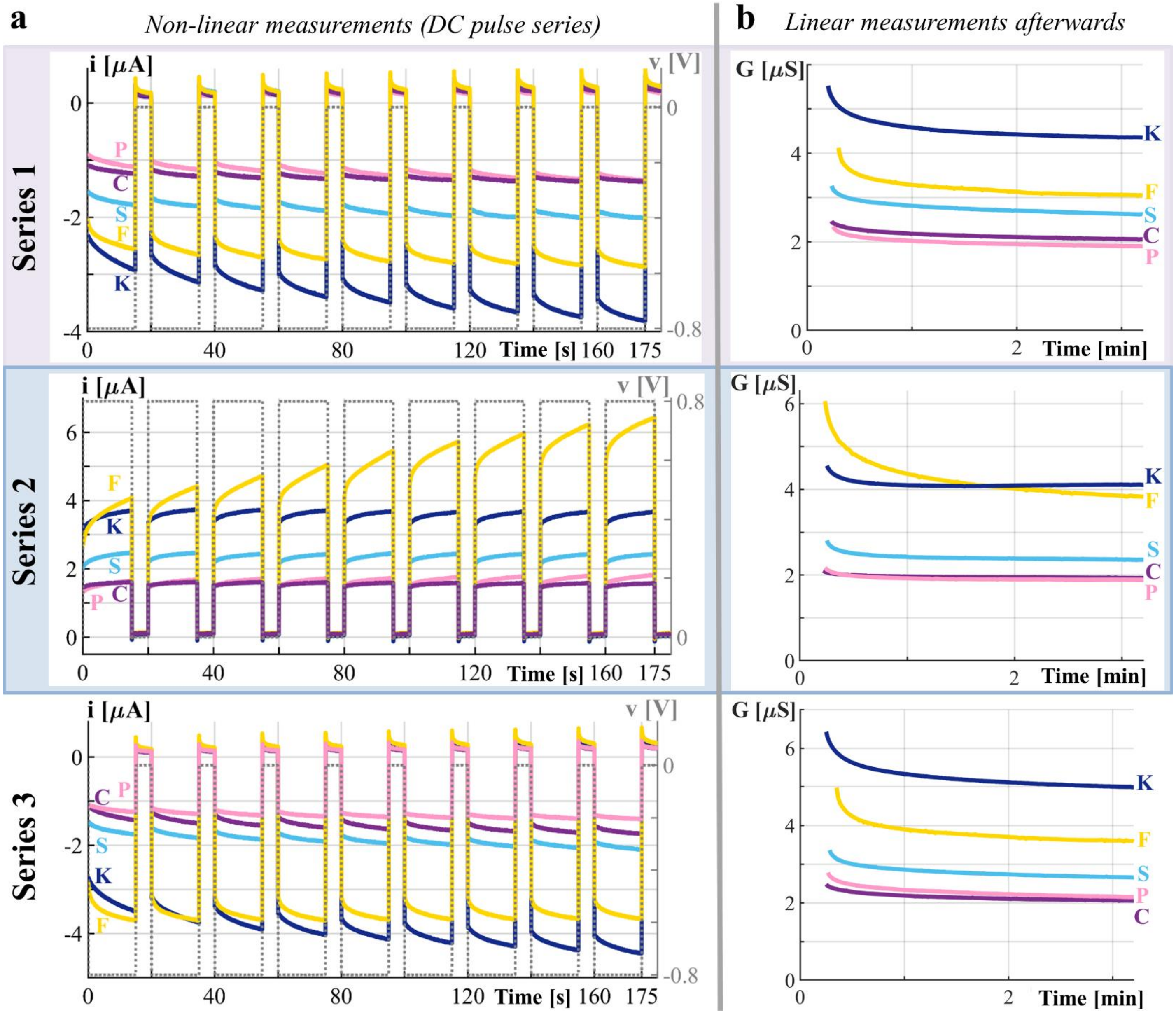

Fig. S2 | Example recordings from the earlobe that represent the stratum corneum memristor shown for subjects C, F, K, P, and S. (**a**) Non-linear measurements with DC pulses. Measured current, *i*, and applied voltage, *v*, plotted over time. The levels of the measured currents in general quite small compared to recordings dominated by the sweat duct memristor. (**b**) Small-signal conductance measurements after each DC pulse series. The time is related to the end of the last pulse of the series. The conductance values of all subjects continuously decrease after all series. The decrease in conductance slows down with time.

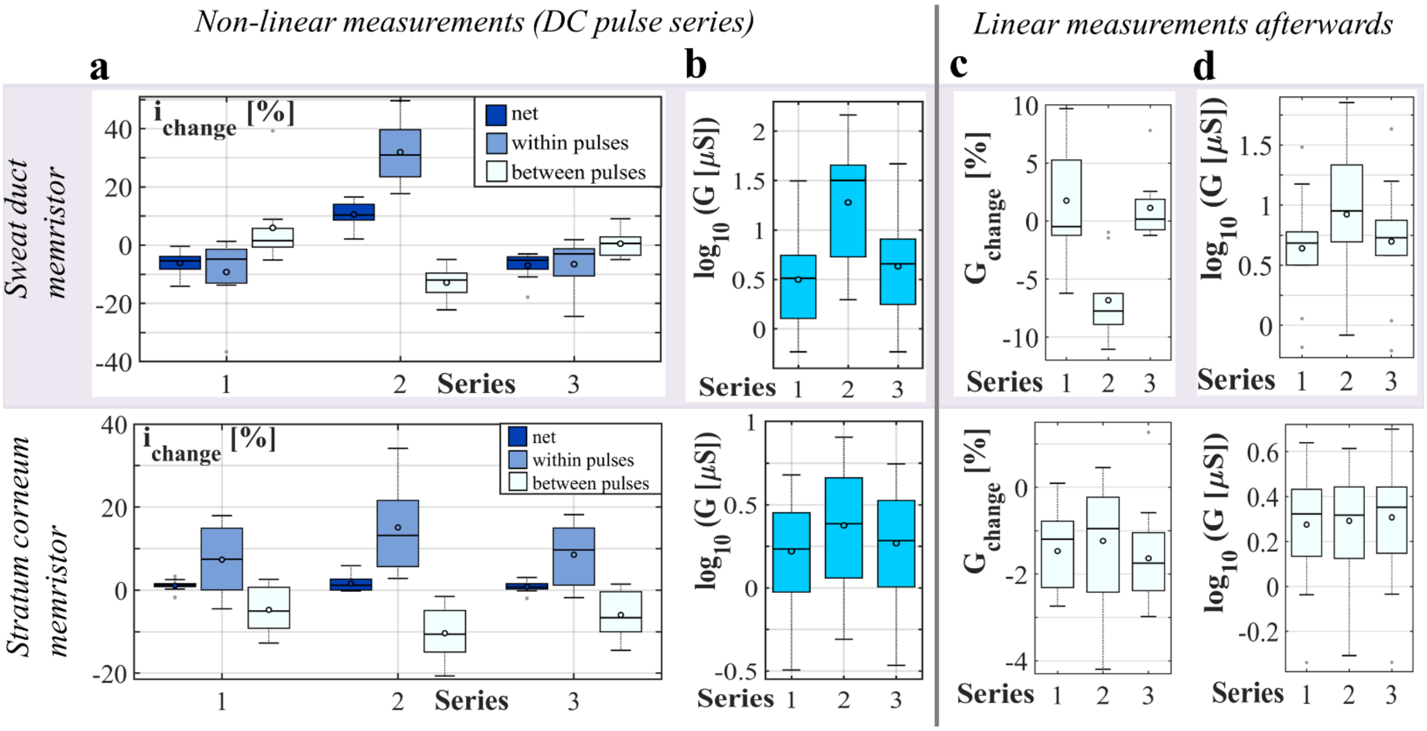

Fig. S3 | Boxplots based on the recordings from the earlobe, separated between the subjects that were dominated by the sweat duct memristor (N=9, top plots) and the subjects that were dominated by the stratum corneum memristor (N=18, plots in the bottom). The recording of one out of 28 subjects was just noise and excluded from this evaluation. The horizontal line in the middle of each boxplot denotes the median; the circle indicates the mean value; and the whiskers indicate the 5% and 95% percentiles. (**a**) Average change in current, *i*, within pulses (within 15 seconds) and between pulses (within 5 seconds) and the net change in current from pulse to pulse, presented as ratios for all three series. (**b**) Memductance *G* (logarithm to base 10) at the end of the last pulse (at 175 s) of each series. (**c**) Changes in small-signal conductance from minute 2 to minute 3 after the last DC pulse. (**d**) Small-signal conductance *G* (logarithm to base 10) 3 minutes after the last pulse. Results from one way repeated measures ANOVA (p-value < 0.001) and pairwise multiple comparisons (Holm-Sidak method) show that the conductance value (logarithm to base 10) in the sweat duct memristor from series 1 differs significantly from that in series 2 but not from that in series 3. There is also a significant difference between the conductance values after series 2 and 3. Results from one way repeated measures ANOVA (p-value < 0.05) and pairwise multiple comparisons (Holm-Sidak method) show that the conductance value (logarithm to base 10) in the stratum corneum memristor from series 1 differs significantly from that in series 3, but there is no significant difference between the conductance values in series 2 and 3 and in series 1 and 2.

Data from the fingertip

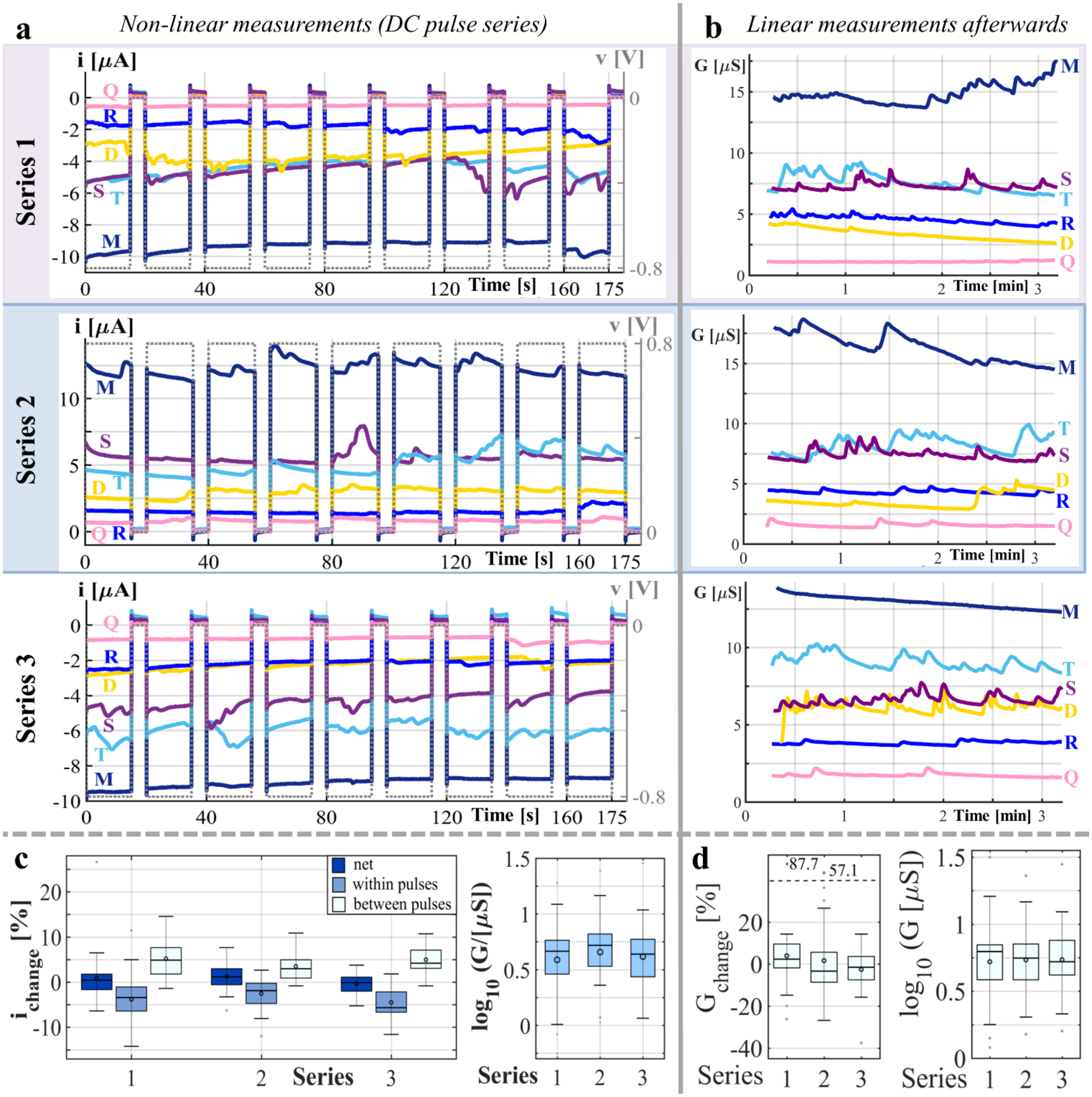

Fig. S4 | Example recordings from the fingertip. (**a**) Measured current, *i*, and applied voltage, *v*, plotted over time for 6 test subjects (D, M, Q, R, S, and T). The behavior at the fingertip differs from that at the earlobe and forehead, since it is not possible to observe a clear trend of the changes in current, and emotional sweating interferes with the measurement. (**b**) Small-signal conductance measurements after each DC pulse series (shown for the same subjects). The time is related to the end of the last pulse of the series. No clear trend of the changes in small-signal conductance can be seen, which differs from the results for the earlobe and forehead, and the recordings have the appearance of normal conductance measurements at an emotionally active skin site. **(c, d**) Boxplots for all subjects (each boxplot is based on the evaluation of *N*=28 subjects). The horizontal line in the middle of each boxplot denotes the median; the circle indicates the mean value; and the whiskers indicate the 5% and 95% percentiles. (**c**) Boxplots related to the DC pulse series. Average change in current, *i*, within pulses (within 15 seconds), between pulses (within 5 seconds) and net change from pulse to pulse, presented as ratios for all three series (left). Memductance *G* (logarithm to base 10) at the end of the last pulse (at 175 s) of each series (right). (**d**) Boxplots related to the small-signal conductance measurements after each pulse series. Change in small-signal conductance from minute 2 to minute 3 after the last DC pulse (left). Small-signal conductance *G* (logarithm to base 10) 3 minutes after the last pulse (right). Results from repeated measures ANOVA of ranks show that there are no significant differences (p-value = 0.65, chi-square = 0.857 with 2 degrees of freedom) in the small-signal conductance values (logarithm to base 10) obtained at minute three among the three series.

Additional data from the forehead

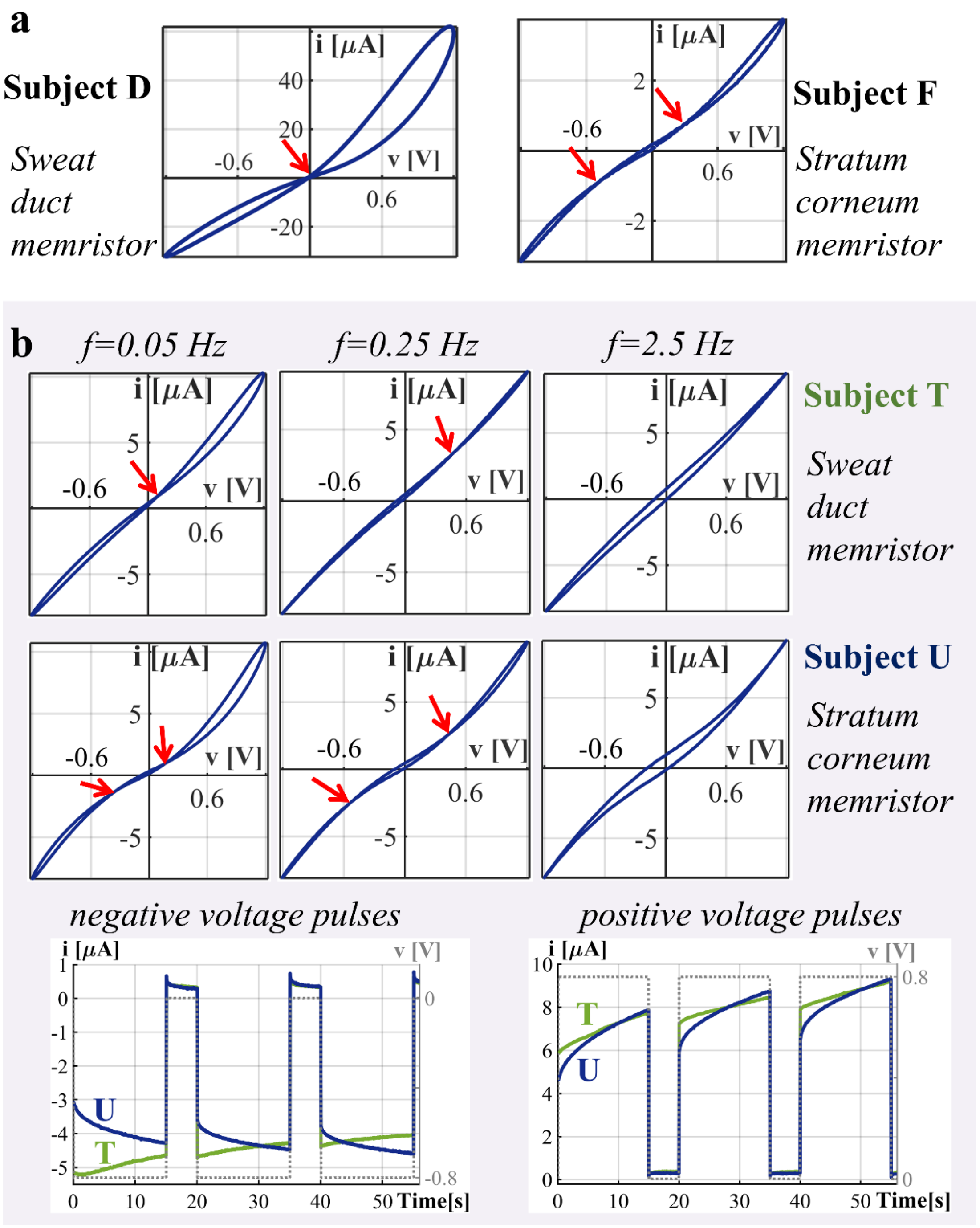

Fig. S5 | Example recordings that demonstrate the difference between the sweat duct memristor and the stratum corneum memristor. (**a**) Voltage-current plots for subjects D and F for applied sinusoidal voltage with 1.2 V amplitude and f=0.05 Hz. These plots are shown in[1] under Creative Commons Attribution 4.0 International License. The arrows indicate the pinched point positions. (**b**) Recordings from the forehead of two additional subjects T and U. The voltage-current plots are shown for three different signal frequencies of applied sinusoidal voltage with amplitude of 1.2 V. The arrows indicate the pinched point positions. Current responses to positive and negative DC voltage pulses (first three pulses of DC pulse series 1 and 2) are shown in the bottom. The recordings of subject U reflect the stratum corneum memristor since two pinched points are observed in the AC voltage current plots and there is a memductance increase independent of the polarity of the applied voltage pulses. The results of subject T imply that the sweat duct memristor dominates that measurements since the AC voltage current plots show one pinched point and it depends on the polarity of the applied voltage pulses whether the memductance decreases or increases. The obtained currents were usually much higher when there was galvanic contacts through the sweat ducts and thus the sweat duct memristor dominated the measurement (see a). However, the here specifically chosen subjects U and T demonstrate that the lower range of the memductance of the sweat duct memristor and the higher range of the memductance of stratum corneum memristor are overlapping. There are subjects in which both memristor types contribute noticeably to the measurement.

References

1 Pabst, O., Martinsen, Ø. G. & Chua, L. O. The non-linear electrical properties of human skin make it a generic memristor. *Scientific Reports* **8**, 15806 (2018).
